# *In vivo *molecular imaging of experimental joint inflammation by combined ^18^F-FDG positron emission tomography and computed tomography

**DOI:** 10.1186/ar3176

**Published:** 2010-11-03

**Authors:** Ingo M Irmler, Thomas Opfermann, Peter Gebhardt, Mieczyslaw Gajda, Rolf Bräuer, Hans P Saluz, Thomas Kamradt

**Affiliations:** 1 Institute of Immunology, Jena University Hospital, Leutragraben 3, 07743 Jena, Germany; 2 Leibniz Institute for Natural Product Research and Infection Biology - Hans-Knöll-Institute, Beutenbergstr. 11a, 07745 Jena, Germany; 3 Institute of Pathology, Jena University Hospital, Ziegelmühlenweg 1, 07743 Jena, Germany; 4 Friedrich-Schiller-University, Fürstengraben 1, 07743 Jena, Germany

## Abstract

**Introduction:**

The purpose of this work was to establish and validate combined small animal positron emission tomography - computed tomography (PET/CT) as a new *in vivo *imaging method for visualisation and quantification of joint inflammation.

**Methods:**

Signalling of radioisotope ^18^F labelled Fluorodeoxyglucose (^18^F-FDG) injected in mice with glucose-6-phosphate isomerase (G6PI)-induced arthritis was analysed by PET/CT. Accumulation of ^18^F-FDG in tissue was quantified by PET measurement, whereas high definition CT delivered anatomical information. The fusion of both images revealed in detail spatial and temporal distribution and metabolism of ^18^F-FDG.

**Results:**

A distinct ^18^F-FDG signal could be measured by PET in carpal and tarsal joints, from mice with early or established arthritis. In contrast, no accumulation of ^18^F-FDG was detectable before arthritis onset. Comparison of ^18^F-FDG joint uptake with histopathological evaluation revealed a significant correlation of both methods.

**Conclusions:**

Small animal PET/CT using ^18^F-FDG is a feasible method for monitoring and, more importantly, quantitative assessment of inflammation in G6PI-arthritis. Since it is possible to perform repeated non-invasive measurements *in vivo*, not only numbers of animals in preclinical studies can markedly be reduced by this method, but also longitudinal studies come into reach, e. g. for individual flare-up reactions or monitoring therapy response in progressive arthritis.

## Introduction

The current gold standard for the assessment of inflammation in small-rodent models of arthritis is histopathological evaluation of joint sections. Apart from semiquantitative grading, limitations of this method include the impossibility of performing *in vivo *or longitudinal studies and the need for relatively large numbers of animals. Non-invasive imaging techniques allowing an objective quantitative assessment of inflammation would, therefore, be a most welcome tool for arthritis research.

Positron emission tomography (PET), predominantly using the radiopharmaceutical ^18^F-labelled fluorodeoxyglucose (^18^F-FDG) as metabolic tracer, is a functional *in vivo *imaging technique that is clinically used mainly for tumor diagnosis, therapy monitoring, and experimental cancer research. ^18^F-FDG is a glucose analog in which the 2'-OH has been replaced by ^18^F. Consequently, ^18^F-FDG cannot be further metabolized after phosphorylation and is trapped and enriched within the cell. This offers the opportunity of a quantifiable ^18^F-FDG PET signal from sites of pathological increased glucose metabolism in the tissue (that is, sites of inflammation). In addition, glucose metabolism is affected by proinflammatory tumor necrosis factor-alpha (TNF-α) and characteristically increased in inflamed tissue [1,2], making PET a potentially interesting technique for the detection and quantification of inflammation. Analyses of radioisotope-labelled FDG uptake *in vitro *revealed a distinct accumulation in fibroblasts and neutrophils, whereas resting macrophages incorporated only small amounts [3]. Stimulation of cells with TNF-α revealed a shift to strong glucose uptake in macrophages and even more in fibroblasts, whereas accumulation in neutrophils remained stable at the level of stimulated macrophages. The accumulation of ^18^F-FDG in cells contributing to synovial inflammation [4-6] could provide a sensitive and non-invasive tool for visualization and quantification of joint inflammation *in vivo*.

In rheumatoid arthritis (RA), only a few studies thus far have examined the use of PET, proposing quantitative ^18^F-FDG PET as a promising technique to measure disease activity and to monitor the efficacy of anti-inflammatory drugs without restriction to morphology-based information [7,8]. PET is also increasingly used in preclinical research [3,9]. Since computed tomography (CT) has a higher resolution than PET, one approach of modern imaging systems is the combination of PET and CT. Thus, in humans, the detailed morphological information provided by CT can be used to localize the anatomical source of PET signals exactly [10].

The goal of our work was to examine, in well-described glucose-6-phosphate isomerase (G6PI)-induced murine arthritis [11-17], whether ^18^F-FDG micro-PET/CT can be used for the quantitative *in vivo *assessment of inflammation in acute and chronic stages of experimental arthritis. To validate the method, results were systematically compared with semiquantitative histopathological analyses. Our findings revealed a linear and statistically significant correlation of ^18^F-FDG PET/CT quantification and histopathological evaluation of inflammatory experimental arthritis. In the field of arthritis research, small-animal PET/CT imaging thus offers new opportunities for a quantitative assessment of inflammation and even can be performed *in vivo*.

## Materials and methods

### Glucose-6-phosphate isomerase-induced arthritis

DBA/1 mice were bred at the animal facility of Jena University (Jena, Germany). All animal studies were approved by the local commission for animal protection (registered number 02-045/08). Arthritis was induced as described elsewhere [11]. In brief, DBA/1 mice were immunized subcutaneously with 400 μg of recombinant human G6PI in emulsified complete Freund's adjuvant (Sigma-Aldrich, Taufkirchen, Germany). Macroscopic evaluation of arthritis was performed as previously described [11]. A score of 0 indicates no macroscopically recognizable signs of arthritis, 1 indicates swelling and redness, 2 means strong swelling and redness, and 3 indicates massive swelling and redness. For the total clinical score per animal, results from all paws were summed. In the experiment assessing practicability of small-animal PET/CT in monitoring therapeutical intervention, 150 μg of soluble tumor necrosis factor receptor (sTNFR) fusion protein (etanercept; Wyeth, Münster, Germany) in 100 μL of saline or saline alone as control was daily administered intraperitoneally, beginning 1 day after immunization.

### Histopathological assessment of glucose-6-phosphate isomerase-induced arthritis

For histopathological examination, mice were sacrificed immediately after PET/CT measurement. Sections of fixed and decalcified joints were evaluated by a pathologist. Arthritis severity of each paw was graded according to a histopathological scoring system, with consideration of acute and chronic inflammatory parameters as described before [18]. In brief, acute inflammation reflects pathological alterations by infiltration of neutrophil granulocytes in the synovial membrane (0 = no, 1 = slight, 2 = moderate, and 3 = severe) and by exudate (0 = no, 1 = slight, 2 = moderate, and 3 = severe), with an additional scoring for the presence of fibrin and the affection of periarticular tissue. The maximal possible value per paw for acute inflammation according to this scoring was 8. Chronic inflammation denotes pathological alterations by synovial hyperplasia, infiltration of mononuclear cells, and fibrosis (0 = no, 1 = slight, 2 = moderate, and 3 = severe), with a maximum of 9. Total inflammation score reflects the sum of acute and chronic inflammation in the joint and periarticular tissue. For individual histopathological arthritis evaluation, scores from all paws were summed.

### Positron emission tomography-computed tomography *in vivo *imaging

A Siemens Inveon small-animal multimodality PET/CT system (Preclinical Solutions; Siemens Healthcare Molecular Imaging, Knoxville, TN, USA) was used for *in vivo *imaging. This PET/CT system is characterized by the combination of two independently operating PET and CT scanners. Radial, tangential, and axial resolutions at the center of the field of view of the PET module are better than 1.5 mm [19,20]. PET acquisitions were carried out with default settings of coincidence timing window of 3.4 ns and energy window of 350 to 650 keV. Images were reconstructed using Fourier rebinning and the two-dimensional ordered-subset expectation maximization (OSEM 2D) algorithm. Attenuation was corrected on the basis of the CT measurements. The CT module consists of a cone beam micro-x-ray source (50-μm focal spot size) and a 2,048 × 3,072 pixel x-ray detector. Our standard micro-CT imaging protocol used 80 kVp at 500 μA, 360° of rotation, and 200 projections per bed position. Micro-CT images were reconstructed using a Shepp-Logan filter and cone-beam filtered back-projection.

Quantitative analysis of ^18^F-FDG accumulation in the period from 40 to 60 minutes after injection was enabled by the image fusion technology of Siemens Inveon Research Workplace software 2.2 (Siemens Healthcare Molecular Imaging). In the kinetic analyses, the 60-minute dataset was divided in 39 time frames; otherwise, data from 40 to 60 minutes after ^18^F-FDG injection were analyzed in a single time frame. Volumes of interest (VOIs) used for quantification of fore and hind limb inflammation were 2.5 and 3.1 mm^3^, respectively. Inflammatory tissue uptake of the glucose analog was measured as percentage of injected dose per gram (% ID/g). Three-dimensional (3D) surface rendering (SR) of areas with increased ^18^F-FDG uptake was carried out with PMOD software 3.0 (PMOD Technologies Ltd., Zurich, Switzerland) by means of SR techniques using an 8% ID/g threshold. Mice were anesthetized with 1.5% to 2% vaporized isoflurane (DeltaSelect, Dreieich, Germany) in oxygen (1.5 L/minute) to prevent animal movement and reduce imaging artefacts. ^18^F (half-life 109 minutes)-labelled FDG (Eckert & Ziegler, Berlin, Germany) with an activity of 9.6 ± 0.6 MBq was injected intravenously in the lateral tail vein.

### Statistical analysis

Statistical differences between groups were evaluated using the non-parametric Mann-Whitney *U *test. Mean scores of fore and hind limbs or individual total scores as the sums of all paws were used for analyses of relationships between histopathological parameters and ^18^F-FDG uptake (Spearman test). Statistical significance was accepted for *P *values of less than 0.05 (*P < 0.05; **P < 0.01; ***P < 0.001). All calculations were performed using the software package SPSS version 16.0 (SPSS Inc., Chicago, IL, USA). In bar charts and text, data are presented as arithmetic mean and standard error of the mean.

## Results

### Histopathological assessment of arthritis severity

**Figure 1:**
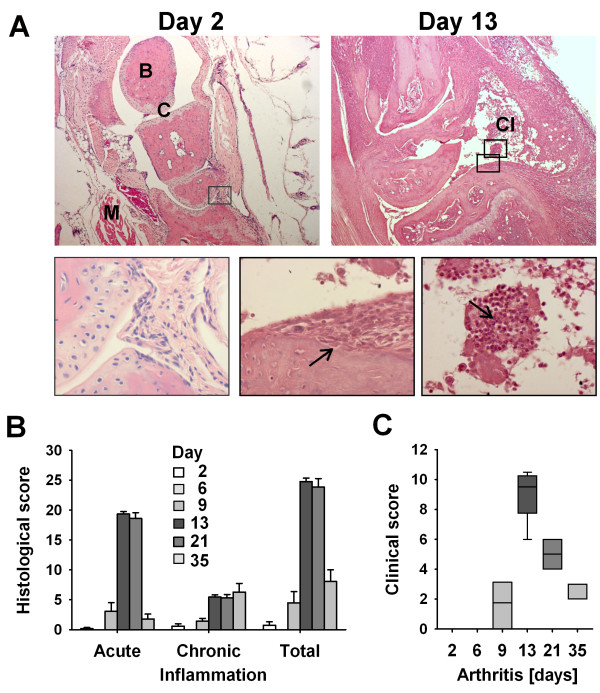
**Histopathological and macroscopical evaluation of glucose-6-phosphate isomerase (G6PI) arthritis severity**. **(a) **Acute murine G6PI-induced arthritis (day 13) provokes a massive accumulation of mainly polymorphonuclear cells (right arrow) and induces erosive processes (left arrow). Hematoxylin-and-eosin staining at original magnifications of ×40 and ×400 is shown. B, bone; C, cartilage; CI, cellular infiltrate; M, muscle. **(b) **Histopathological semiquantitative assessment of paw sections revealed a maximum of acute inflammation 13 and 21 days after arthritis induction, whereas chronic inflammation parameters were present past day 13. Total inflammation as the sum of acute and chronic parameters was at a maximum at days 13 and 21 and decreased at day 35 (*n *= 6 to 9 per time point). **(c) **Macroscopic scoring of paw swelling showed a maximum of inflammation at day 13 after experimental arthritis induction and gradual decreases at days 21 and 35.

To examine the usability of state-of-the-art small-animal PET/CT molecular imaging as an objective method to quantify inflammation in experimental arthritis research, severity of inflammation was analyzed at six different time points (days 2, 6, 9, 13, 21, and 35) in the course of G6PI-induced arthritis, using both micro-PET/CT and histology. Histopathological assessment of the degree and components of immune cell infiltration, synovial hyperplasia, and exudate in arthritic joints enabled a semiquantitative scoring of experimental arthritis severity (Figure 1a). Histology revealed no pathological changes in joints and periarticular joint tissue 2 and 6 days after G6PI immunization (Figure 1b). At day 9, histopathological signs of inflammation coincided with the onset of clinical arthritis. Total individual scores as the sum of acute and chronic parameters revealed a maximum of arthritis severity at days 13 (24.8 ± 0.6) and 21 (23.9 ± 1.4) and a subsequent decrease at day 35 (8.1 ± 1.9). Scoring of macroscopically visible signs of arthritis (redness and swelling) showed a similar course of experimental arthritis severity (Figure 1c). The first clinical signs appeared at day 9 (1.7 ± 0.6), and the maximal clinical score (8.9 ± 0.5) was obtained at day 13. Clinical signs of arthritis were attenuated at day 21 (4.9 ± 0.4) and day 35 (2.4 ± 0.2).

### Small-animal positron emission tomography/computed tomography molecular imaging

**Figure 2:**
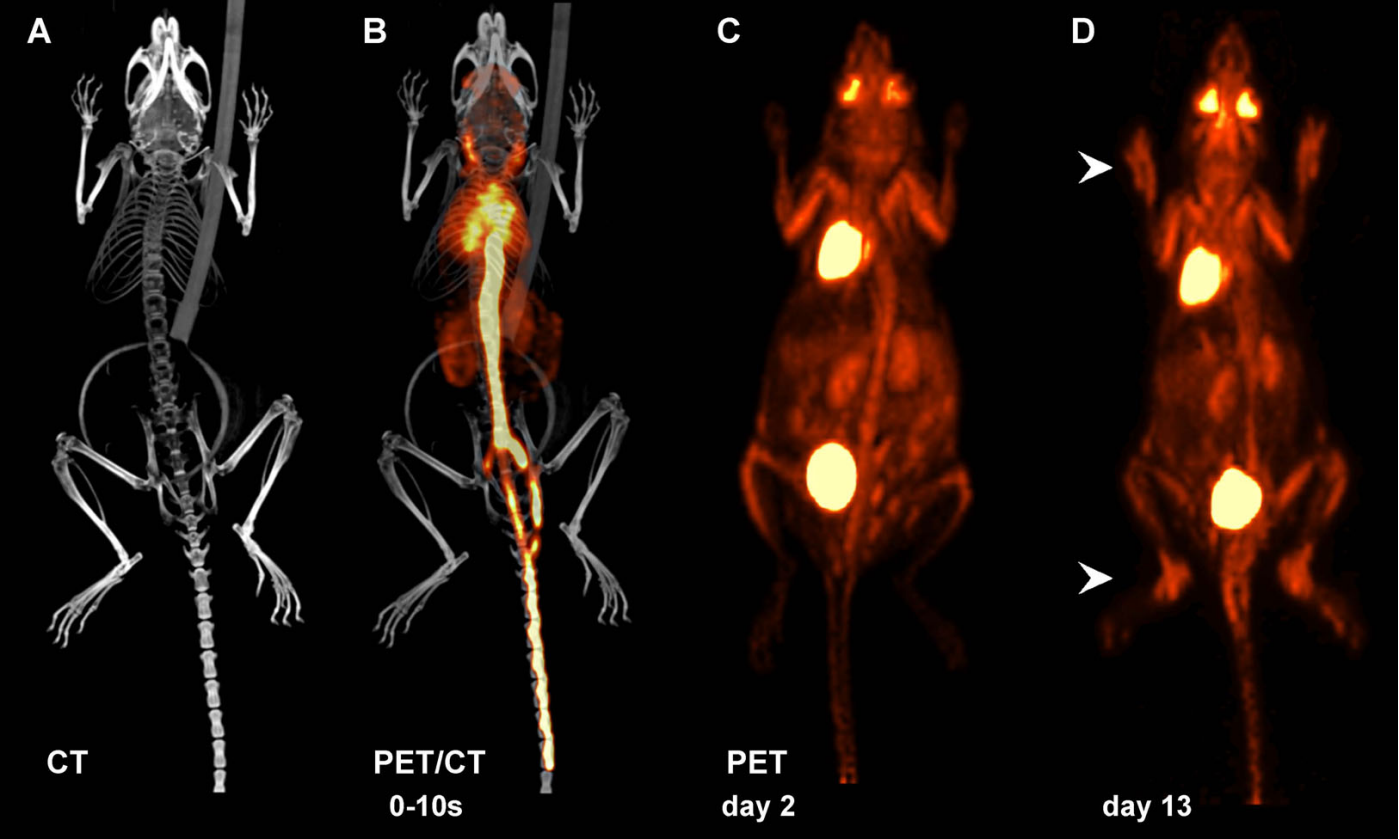
**^18^F-labelled fluorodeoxyglucose (^18^F-FDG) positron emission tomography/computed tomography (PET/CT) imaging in experimental arthritis**. **(a) **High-resolution CT provided detailed anatomical information of skeletal elements for accurate allocation of PET signalling. **(b) **Rapid ^18^F-FDG biodistribution via blood circulation in a time frame 0 to 10 seconds after tail vein injection, visualized by PET and CT image fusion. **(c) **PET imaging of mice before arthritis onset (day 2) revealed enrichment of ^18^F-FDG in tissue and organs (in particular, heart, bladder, kidneys, and eyes). **(d) **Acute inflammatory arthritis (day 13) caused additional accumulation of ^18^F-FDG at sites of pathological increased glucose metabolism (that is, inflamed carpal and tarsal joints) (arrowheads).

Scanning of mice with micro-PET/CT generated high-resolution 3 D images with information on temporal and spatial biodistribution of ^18^F-FDG, reflecting the intensity of tissue glucose metabolism. Precise anatomical localization of VOIs was given by high-resolution CT imaging (Figure 2a). The combination of PET and CT data enabled detailed visualization of ^18^F-FDG signalling in the body (Figure 2b). ^18^F-FDG reached heart and lung via the *vena cava *within the first 0- to 10-second time frame after injection and subsequently was distributed in the gastrointestinal tract and cranially via the carotid arteries. Before arthritis onset (day 2), tracer enrichment was restricted to tissue with high basal metabolic activity (in particular, heart muscle and eyes) and organs of the excretory system (kidneys and bladder; Figure 2c; Additional file 1). In acute experimental arthritis at day 13, additional hot spots of ^18^F-FDG signalling could be detected in the joints of fore and hind paws, indicating a specific ^18^F-FDG uptake in inflamed joints (Figure 2d; Additional file 2).

### Accumulation of ^18^F-FDG in joints

**Figure 3:**
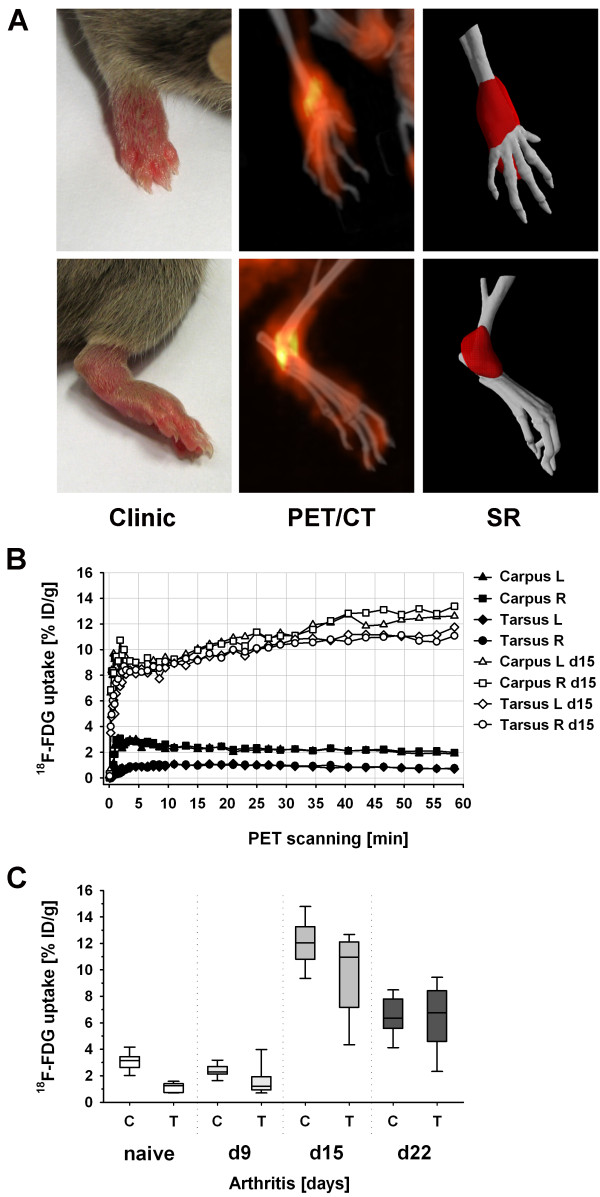
**Accumulation of ^18^F-labelled fluorodeoxyglucose (^18^F-FDG) in paws of DBA/1 mice with glucose-6-phosphate isomerase (G6PI)-induced arthritis**. **(a) **Clinical polyarthritis is characterized by swelling and redness of paws. Enhanced glucose metabolism of inflamed tissue caused an increased uptake and subsequent enrichment of ^18^F-FDG in carpal and tarsal joints of arthritic mice, visualized by high-resolution positron emission tomography/computed tomography (PET/CT) imaging. Surface-rendering (SR) of data using an 8% injected dose per gram threshold allowed three-dimensional visualization of tissue with pathological increased glucose metabolism. **(b) **Quantification of accumulated ^18^F-FDG as a percentage of injected dose per gram (% ID/g) within 0 to 60 minutes after injection. ^18^F-FDG enrichment over time in left (L) and right (R) carpal and tarsal joints in an exemplary mouse before immunization with G6PI (black symbols) (that is, before pathological changes occurred) and increased ^18^F-FDG joint accumulation in the same animal at the stage of acute inflammatory G6PI-induced arthritis at day 15 after immunization (white symbols) are shown. **(c) **Repeated ^18^F-FDG PET/CT *in vivo *determination and quantification of disease severity in carpal (C) and tarsal (T) joints in the same mice 2 days before and 9, 15, and 22 days after arthritis induction (*n *= 5).

Swelling and erythema of paws are characteristic clinical features of acute G6PI-induced arthritis (Figure 3a). ^18^F-FDG accumulation measured by micro-PET/CT revealed hot spots of inflammatory metabolic activity in wrist and ankle joints and a less intense inflammation of smaller distal joints. SR of PET/CT data using thresholds (shown is 8% ID/g, according to the level of ^18^F-FDG joint accumulation in acute G6PI arthritis) enabled a fusion-based 3 D visualization of morphological structures and regions, where ^18^F-FDG was enriched because of inflammatory processes. Kinetic analysis of ^18^F-FDG signalling in joints before G6PI immunization (Additional file 3) and at the stage of acute inflammatory arthritis (day 15; Additional file 4) revealed a beginning of ^18^F-FDG accumulation approximately 5 minutes after injection (Figure 3b). In arthritic joints, ^18^F-FDG uptake was still slightly increasing 60 minutes after tracer injection. In healthy joints, negligible tracer enrichment occurred during the initial 20 minutes after injection, followed by a slight decline. In arthritic animals, percentages of injected radioisotope activities in the joint regions were between 8% and 15% ID/g, whereas tracer uptake in the same joints was around 1% to 3% ID/g before immunization. Therefore, differences in ^18^F-FDG uptake of arthritic and healthy joints were pronounced, could be attributed to joint tissue glucose metabolism changes caused by pathogenesis of G6PI-induced arthritis, and could easily be quantified by small-animal PET/CT techniques.

**Figure 4:**
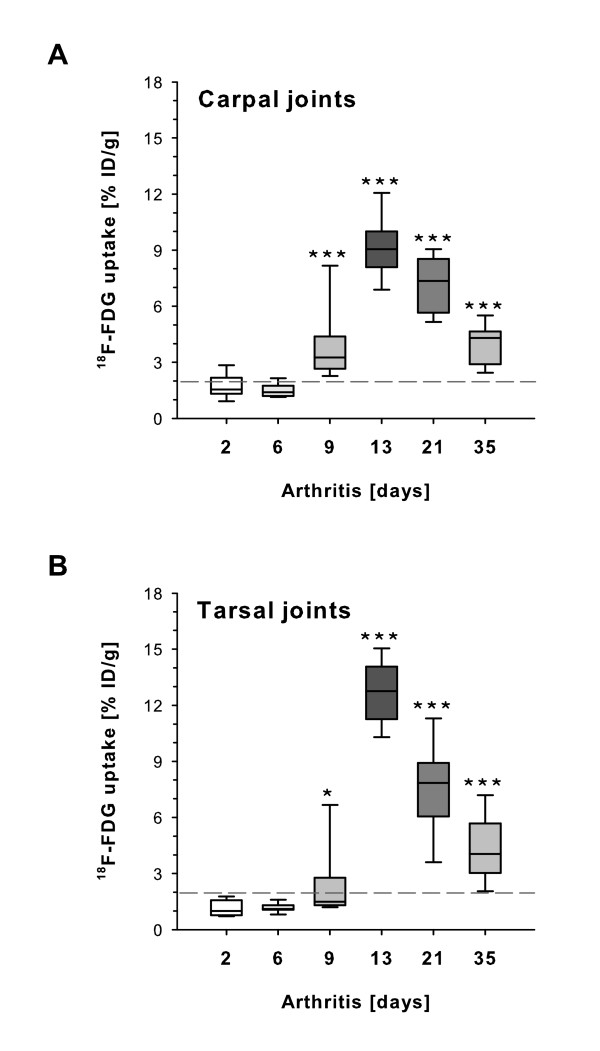
**Quantification of ^18^F-labelled fluorodeoxyglucose (^18^F-FDG) joint uptake in progressive arthritis**. Inflammatory metabolic effects of arthritis differing from ^18^F-FDG accumulation in normal joint tissue are seen above an arbitrary baseline of 2% of injected dose per gram. **(a) **Onset of clinical arthritis at day 9 resulted in a significant increase of ^18^F-FDG signalling in the fore limb carpal joints with a maximum at day 13 and a subsequent decreased ^18^F-FDG uptake in parallel to declining inflammation at days 21 and 35 (*n *= 6 to 9 per time point). **(b) **Hind limb arthritis progression reflected by ^18^F-FDG signalling in the tarsal joint, coinciding with carpal joint signalling. % ID/g, percentage of injected dose per gram. *P < 0.05; **P < 0.01; ***P < 0.001.

Repeated carpal and tarsal inflammation assessment in the same animals by ^18^F-FDG PET measurements at four time points (unimmunized and days 9, 15, and 22) did not influence the course of experimental arthritis (Figure 3c). Both the clinical scores (data not shown) and the levels of carpal and tarsal ^18^F-FDG joint uptake (Figures 3c and 4) were similar in those mice that underwent small-animal PET/CT examination repeatedly and those that were examined by PET/CT only once and then employed for histopathological evaluation of arthritis.

### *In vivo *quantification of joint inflammation by ^18^F-FDG positron emission tomography/computed tomography

To analyze the usefulness of micro-PET/CT imaging for monitoring severity of inflammatory arthritis, ^18^F-FDG uptake in paws from a total of 42 mice at 6 different time points of experimental disease was quantified. In joint tissue without pathological changes (days 2 and 6), mean accumulation of ^18^F-FDG in fore (Figure 4a) and hind (Figure 4b) limbs was below 2% ID/g, the level of ^18^F-FDG accumulation over time in joints of healthy animals before induction of experimental disease (Figure 3b). At the onset of clinical arthritis at day 9, emerging inflammation was associated with an increase of glucose metabolism and induced a significant increase of ^18^F-FDG uptake compared with day 2 (carpal: *P *= 0.0002; tarsal: *P *= 0.02). Mean values of % ID/g were 3.8 ± 0.57 in carpal and 2.4 ± 0.55 in tarsal joints. ^18^F-FDG uptake was further increased at the peak of acute arthritis at day 13 (carpal: 9.2 ± 0.38; tarsal: 12.7 ± 0.40), followed by subsequent decreases at day 21 (carpal: 7.1 ± 0.39; tarsal: 7.6 ± 0.66) and day 35 (carpal: 3.9 ± 0.29; tarsal: 4.4 ± 0.46). Hence, PET data revealed a 4- to 10-fold increase of ^18^F-FDG joint uptake in inflammatory stages of arthritis. Interestingly, ^18^F-FDG uptake revealed significant (*P *= 0.001; Figure 4a,b) deviating metabolic activity between days 13 and 21, demonstrating a reduced inflammatory activity at day 21, whereas histopathological assessment showed no difference of arthritis severity in these groups (*P *= 0.7; Figure 1b). This reduced inflammation activity measured by PET/CT at day 21 was confirmed by macroscopical arthritis assessment (*P *= 0.001; Figure 1c).

### Histopathological evaluation correlates significantly with positron emission tomography quantification

**Figure 5:**
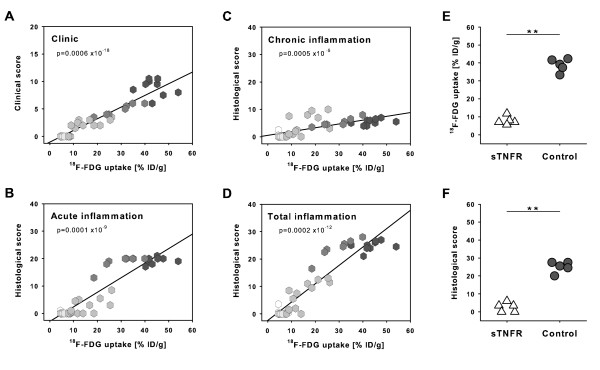
**Coherence of ^18^F-labelled fluorodeoxyglucose (^18^F-FDG) uptake and clinical and histopathological assessment of inflammatory arthritis and use of ^18^F-FDG positron emission tomography/computed tomography for evaluation of therapeutical intervention**. **(a) **Clinical assessment of experimental arthritis was correlated with ^18^F-FDG enrichment at days 2, 6, 9, 13, 21, and 35 after arthritis induction (*n *= 42). **(b) **Regression analysis of acute inflammation and ^18^F-FDG uptake evidenced a significant correlation of both parameters. **(c) **Chronic inflammation also significantly correlated with ^18^F-FDG signalling. **(d) **Histopathological scoring of total inflammation as the sum of acute and chronic inflammation showed a significant correlation with ^18^F-FDG uptake. **(e) **^18^F-FDG uptake as a measure of inflammatory arthritis activity in arthritic mice treated with soluble tumor necrosis factor receptor (sTNFR) (etanercept) is significantly reduced compared with untreated controls in the acute stage of arthritis (**P = 0.009; *n *= 5 per group). **(f) **Histopathological assessment of diminished (**P = 0.008) arthritis severity in the same mice coincided with ^18^F-FDG uptake. % ID/g, percentage of injected dose per gram.

To validate the method and to determine whether ^18^F-FDG uptake was correlated with histopathological changes in arthritis, we performed correlation analysis of individual quantitative PET results with semiquantitative clinical and histopathological scoring data. Individual ^18^F-FDG PET/CT quantification of inflammation severity correlated significantly with macroscopic clinical scoring (Figure 5a). Furthermore, there were significant correlations of ^18^F-FDG uptake and histopathological data assessment regarding scoring of acute, chronic, and total inflammation (Figure 5b-d). Animals that scored in the upper range of the histopathological arthritis index also achieved maximum values in PET analysis. Interestingly, arthritis severity in animals with maximal histopathological scoring was further differentiated by ^18^F-FDG PET/CT technique. This means that 25% of the animals with the highest histopathological scores ranged in the top 12% of the total bandwidth of histopathological scoring. In contrast, assessment of inflammation by PET/CT enabled a more refined staging. Here, the 25% of the animals that showed the strongest ^18^F-FDG accumulation in the joints covered the top 38% of the total range of ^18^F-FDG uptake. For carpal and tarsal joints and for joints total, correlations of ^18^F-FDG uptake and histopathological assessment were highly significant (Table 1). These significant correlations of both methods demonstrate that small-animal PET/CT is a reliable imaging method for *in vivo *quantification of inflammatory arthritis.

**Table 1:** Statistical analysis of correlations of ^18^F-FDG joint uptake referred to histopathological and clinical evaluation of inflammatory arthritis (Spearman test)

	^18^F-FDG uptake, percentage of injected dose per gram					
	
	Carpal joints		Tarsal joints		Joints total	
			
	Correlation	*P *value	Correlation	*P *value	Correlation	*P *value
Histological score						
Inflammation						
Acute	0.841	< 0.0001^a^	0.857	< 0.0001^a^	0.842	< 0.0001^a^
Chronic	0.697	< 0.0001^a^	0.796	< 0.0001^a^	0.740	< 0.0001^a^
Total	0.903	< 0.0001^a^	0.904	< 0.0001^a^	0.894	< 0.0001^a^
Clinical score	0.938	< 0.0001^a^	0.935	< 0.0001^a^	0.929	< 0.0001^a^

^a^Highly significant correlations. ^18^F-FDG, ^18^F-labelled fluorodeoxyglucose.

### ^18^F-FDG positron emission tomography/computed tomography and histopathological assessment in monitoring therapeutic intervention

Experimental modulation of inflammation is an important aim of arthritis research. To examine the usability of small-animal PET/CT to monitor the outcome of a therapeutic intervention, G6PI-immuized mice were injected with human sTNFR (etanercept) or saline. We quantified ^18^F-FDG enrichment in the joints of treated and control mice by PET/CT and found a significant 4.9-fold decrease of total ^18^F-FDG uptake in sTNFR (etanercept)-treated arthritic mice (Figure 5e). Comparable results were obtained using histopathological assessment of therapeutic intervention (Figure 5f). The correlation coefficient of histopathological assessment and ^18^F-FDG uptake was 0.862, whereas a *P *value of less than 0.002 revealed statistical significance of correlation of both methods. Thus, PET/CT is a convenient technique for monitoring treatment modalities in experimental arthritis, even with small numbers of animals.

## Discussion

PET data have been shown to correlate with clinical and sonographic parameters in studies on patients with RA [7,8]. The use of *in vivo *imaging techniques, including PET, in murine models of arthritis has been hindered by the reduced dimensions of the subjects of study. Here, we used a small-animal PET/CT system to assess arthritis severity in mice. This combination of PET with high-definition CT in a single device allows the exact 3 D anatomical localization of PET signals (that is, ^18^F-FDG accumulation in the tissue). Moreover, combined PET and CT imaging also allows the use of CT data to correct for tissue attenuation of PET signals. Our findings demonstrate the feasibility and usefulness of ^18^F-FDG joint uptake for objective *in vivo *quantification of synovial inflammation in the course of G6PI-induced arthritis. Whereas histopathological evaluation inevitably requires sacrificing animals and therefore restricts the complete data range of individual arthritis progression to a single snapshot, ^18^F-FDG PET/CT allows repeated investigations of the same subjects. This not only drastically reduces the number of animals required for a study but also offers the appealing possibility of longitudinal analyses within the same animal. Furthermore, *in vivo *imaging increases statistical quality of data in treatment studies because the status of an animal before and after disease-modulating intervention can be compared directly. Therefore, combined PET/CT using ^18^F-FDG as a tracer is a new and powerful tool to quantify experimental joint inflammation non-invasively and accurately *in vivo*.

Another non-invasive technique, thermal signature analysis, has been used to analyze rat rather than mouse arthritis models [21]. Gait analysis, which has been performed in both rat and mouse models, provides indirect but not direct measurement and quantification of inflammation [22,23]. The reduced need to sacrifice animals for preclinical arthritis studies, the option to perform intra-individual longitudinal studies, and the fact that quantitative evaluation of inflammation is obtained faster with PET/CT than with histopathological examinations all encourage the use of PET/CT.

In contrast to histopathology, ^18^F-FDG PET/CT cannot reveal the exact cellular composition of inflammatory infiltrates. However, the necessity to acquire this information depends upon the specific question of the study and therefore is only casually addressed in experimental arthritis studies in detail. Moreover, this shortcoming of ^18^F-FDG PET/CT is compensated by functional knowledge and an increased range of values. First, histopathological assessment gives information about the number of cells present at the site of inflammation, whereas ^18^F-FDG PET/CT gives information about metabolic activation of these cells. This difference facilitates the early detection of arthritis resolution. In our experiments, this is exemplified by the deviance in G6PI arthritis assessment at day 21 by using histology or PET/CT and macroscopical evaluation. Second, even among mice with maximal scores in semiquantitative histopathological assessment, ^18^F-FDG PET/CT still showed quantitative differences and thus enhanced discernability. Another aspect of ^18^F-FDG is the reduction of methodological variability since approval as a nuclear medicinal product for humans guarantees a constant quality of the agent and therefore reproducibility of data.

In regard to CT images in small-animal PET/CT hybrid systems, only a small technological step in image resolution is necessary to use CT data not only for gaining anatomical information but, beyond that, to assess inflammation and erosive processes in a single *in vivo *data acquisition, extending the usefulness of this sophisticated molecular imaging technique. The use of PET/CT is not restricted to ^18^F-FDG or glucose metabolism. In human autoimmune diseases, the approaches of other imaging techniques, in particular scintigraphy, using radiolabelled antibodies or drugs for staging and therapy decision-making, increasingly attracted interest [24,25]. Therefore, other forms of small-animal PET/CT application worth further developmental work are localizing targets of therapeutical monoclonal antibodies *in situ*, if these were labelled with radioisotopes, and the possibility of revealing the presence or expression level of specific receptors correlated with disease in inflammatory regions [26-28].

## Conclusions

In the current studies, we evaluated, for the first time, the use of combined ^18^F-FDG PET/CT for *in vivo *quantification of inflammatory conditions in experimental murine arthritis. Robust statistical analyses at various time points of arthritis progression showed a highly significant correlation to histopathological inflammation assessment, proposing ^18^F-FDG small-animal PET/CT as a feasible tool for preclinical arthritis research. In addition to monitoring efficacy of anti-inflammatory drugs, it offers the possibility of performing large-scale animal studies including repeated *in vivo *examinations. Next to objective quantification of inflammation, information about synovial metabolic activity, and an increased discernability of individual disease severity, this is a major advantage of this new and promising imaging technique, enabling a considerable contribution to the claim of reducing animal numbers in experiments.

## Abbreviations

% ID/g: percentage of injected dose per gram; 3D: three-dimensional; ^18^F-FDG: ^18^F-labelled fluorodeoxyglucose; CT: computed tomography; G6PI: glucose-6-phosphate isomerase; PET: positron emission tomography; RA: rheumatoid arthritis; SR: surface rendering; sTNFR: soluble tumor necrosis factor receptor; TNF-α: tumor necrosis factor-alpha; VOI: volume of interest.

## Competing interests

The authors declare that they have no competing interests.

## Authors' contributions

IMI helped to establish imaging techniques, perform the experiments, analyze data, design the studies, interpret data, and draft the manuscript. TO and PG helped to establish imaging techniques, perform the experiments, and analyze data. MG conducted the histopathological assessment. RB and HPS participated in data interpretation and critically reviewed the manuscript. TK helped to design the studies, interpret data, and draft the manuscript. All authors read and approved the final version of the manuscript.

## Supplementary Material

Additional file 1 **^18^F-FDG joint accumulation before onset of experimental arthritis (day 2)**. Injected ^18^F-FDG was enriched in tissues with a high basal glucose metabolism and in organs of the excretory system (heart muscle, eyes, bladder and kidneys), but not in extremities. Three dimensional view of ^18^F-FDG signalling 60 min post tracer injection. AVI video format. Click here for file

Additional file 2 **^18^F-FDG joint accumulation in acute experimental arthritis (day 13)**. Onset of experimental arthritis induced distinct ^18^F-FDG enrichment in joint regions affected by inflammation. Three dimensional view of ^18^F-FDG signalling 60 min post tracer injection. AVI video format. Click here for file

Additional file 3 **Kinetic of ^18^F-FDG uptake before onset of experimental arthritis**. Tail vein injected tracer was rapidly distributed in the whole body and subsequently accumulated in the heart muscle and in the bladder. Coronal view of ^18^F-FDG signalling 0-60 min post injection. AVI video format. Click here for file

Additional file 4 **Kinetic of ^18^F-FDG uptake in acute experimental arthritis**. Besides tracer accumulation in the heart muscle and in the bladder, under inflammatory conditions carpal and tarsal joints were additional hot spots of ^18^F-FDG enrichment. Coronal view of ^18^F-FDG signalling 0-60 min post injection. AVI video format. Click here for file
